# Effect of cyprodinil and fludioxonil pesticides on bovine liver catalase activity

**DOI:** 10.1080/13102818.2014.992740

**Published:** 2015-01-07

**Authors:** Hasan Karadag, Fadil Ozhan

**Affiliations:** ^a^ Department of Chemistry, Faculty of Science and Letters, University of Adiyaman, Adiyaman, Turkey

**Keywords:** cyprodinil, fludioxonil, inhibition, pesticide, catalase

## Abstract

This study investigated if the use of the pesticides cyprodinil and fludioxonil produced an inhibitory effect on the bovine liver catalase (CAT) activity. It was documented that the activity of the enzyme decreased with increasing concentrations of cyprodinil and fludioxonil from 0 to 500 ppm. At pesticide concentrations of 250 and 500 ppm, the activity of CAT remained unchanged and passed to a steady state. The exposure to cyprodinil in concentrations of 10, 50, 100, 250 and 500 ppm, led to a decrease in the per cent of the CAT enzyme activity calculated as 45.4, 68.0, 73.0, 77.8 and 77.4, respectively. Similarly, the exposure to fludioxonil in concentrations of 10, 50, 100, 250 and 500 ppm, produced the following percentage decrease in the CAT enzyme activity: 20.0, 30.8, 42.8, 46.3 and 45.9, respectively. Cyprodinil inhibited CAT competitively, whereas the mechanism of fludioxonil inhibition over the enzyme was non-competitive.

## Introductıon

Catalase (CAT) is a general enzyme found in nearly all living organisms exposed to oxygen. CAT is an enzyme with antioxidant properties. It catalyzes the degradation of hydrogen peroxide to water and oxygen.[1] The molecule of CAT is made of a tetramer of four polypeptide chains, each over 500 amino acids long. CAT contains four porphyrin heme (iron) groups that permit the enzyme to react with hydrogen peroxide.[2] Hydrogen peroxide itself is a harmful co-product of many general metabolic processes. In order that damage to cells and tissues is avoided, the produced hydrogen peroxide must be immediately converted into other, less-reactive substances. For this purpose, CAT is often used by cells to quickly catalyze the degradation of hydrogen peroxide into less-reactive oxygen and water molecules.[3]

The fungicides cyprodinil and fludioxonil are active ingredients of the product Switch 62.5WG. It contains 37.5% cyprodinil and 25.0% fludioxonil.[4] The product is used for the control of *Botrytis cinerea* (Grey Mold). Cyprodinil, is a widespread pyrimidinamine fungicide, with a worldwide agricultural usage for protection of fruit plants, vines, cereals and vegetables from a large number of pathogens.[5] Fritz et al.,[6] Kanetis et al.,[7] Masner et al. [8] and Mindt [9] reported that cyprodinil acts by inhibiting the biosynthesis of methionine and other thionic amino acids in the fungi. Fludioxonil, is a phenylpyrrole antifungal compound derived from the antibiotic pyrrolnitrin.[10] It has a wide antifungal spectrum and is now used to control a variety of important plant-pathogenic fungi. It is a unique antifungal compound that targets signal transduction.[11]

In this study, the optimal temperature of CAT was determined and the inhibition of CAT by the two pesticides, cyprodinil and fludioxonil, was observed.

## Materials and methods

### Chemicals

CAT from bovine liver (C-1345) was obtained from Sigma. Fludioxonil (4-(2,2-difluoro-1,3-benzodioxol-4-yl) pyrrole-3-carbonitrile) (46102) and cyprodinil (4-cyclopropyl-6-methyl-N-phenylpyrimidin-2-amine) (34389) were obtained from Fluka Riedel-de Haen. All other used chemicals were of analytical grade.

### CAT activity measurement

CAT activity was measured according to the methods described by Lartillot et al.[12]

### Protein measurement

The protein concentration was determined using the method of Lowry et al.[13]

### Effects of pesticides on enzyme activity

A total of 5000 ppm cyprodinil and fludioxonil were prepared by us and dissolved in 2 ml ethyl alcohol. Following, aliquots were arranged to match 10, 50, 100, 250 and 500 ppm, and then each of these was mixed with 700 µL enzyme solution. The final volume of enzyme solution plus ethyl alcohol and pesticide was 1 ml. The mixture was incubated at room temperature for an hour and then the activities of CAT were measured.

### Temperature optimum

Activities of CAT were measured at temperatures between 5 and 50 °C and the optimal temperature was determined.

### pH optimum

A phosphate buffer (50 mM, pH 7.5) was used according to Tukel and Alptekin in order to determine the optimal pH for the reaction.[14]

### Data analysis

Data were presented as mean ± standard error. For the statistical analyses, one-way analysis of variance was used, followed by the Student Newman–Keul's test using the SPSS version 21 statistical software (SPSS Inc., Chicago, IL, USA). Differences were considered significant, if *P* < 0.05.

## Results and discussion

### Temperature optimum

**Figure 1.**
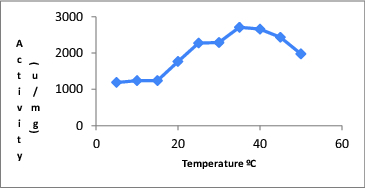
Effect of temperature on catalase activity.

The maximal activity of CAT was determined at 35 °C, which is also presented in Figure 1.

### Exposure of CAT to cyprodinil

At different concentrations of cyprodinil mixed with the CAT enzyme, its activities were measured and were given in Table 1. As cyprodinil concentrations increased, CAT was inhibited and its activity decreased. There were statistically significant differences between all determined values for the inhibited CAT, which interacted with the cyprodinil at the mentioned concentrations (*P* < 0.05, *n* = 3), and the control activity. The calculated per cent decrease of CAT enzyme activity after exposure to cyprodinil at concentrations of 10, 50, 100, 250 and 500 ppm, were as 45.4, 68.0, 73.0, 77.8 and 77.4, respectively.

**Table 1.** Effect of cyprodinil concentrations on catalase activity.

Cyprodinil concentration (ppm)	Activity ± standard error (U/mg)
0	797 ± 2 **a**
10	435 ± 4 **b**
50	255 ± 9 **c**
100	215 ± 2 **d**
250	177 ± 5 **e**
500	180 ± 4 **e**

Values are expressed as mean ± standard error (*n* = 3). In the table, ‘a, b, c, d and e’ letters were used for differences of activity levels. There were statistical differences between the data which were shown with different letters (*p* ˂ 0.05).

### Inhibition type of cyprodinil to CAT

**Figure 2.**
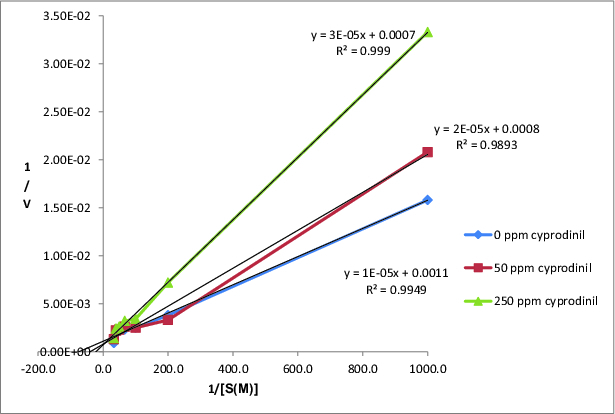
Lineweaver–Burk graph of CAT exposed with cyprodinil.

The activities of CAT were determined at different substrate concentrations of H_2_O_2_ (1, 5, 10, 15, 20, 25 and 30 mM) and at different concentrations of cyprodinil (50 and 250 ppm). Lineweaver–Burk graph of CAT was drawn by using the obtained results (Figure 2).

According to Figure 2, V_max_, K_m_ and K_i_ (inhibition constant) of CAT at different concentrations of cyprodinil were calculated and were shown in Table 2. The V_max_ values determined by us were near each other at the Lineweaver–Burk graph and we found increased K_m_ values. Thus, it is suggested by us that cyprodinil acted on the activity of CAT via a competitive type of inhibition.

**Table 2.** Effect of cyprodinil and fludioxonil on the kinetic parameters of CAT.

Pesticides concentrations	V_max_ (U/mg)	K_m_ (M H_2_O_2_)	K_i_ (M)
0 ppm cyprodinil	919	1.35 × 10^−2^	0.00
50 ppm cyprodinil	1263	2.50 × 10^−2^	1.65 × 10^−7^
250 ppm cyprodinil	1405	4.58 × 10^−2^	5.00 × 10^−7^
0 ppm fludioxonil	1356	2.88 × 10^−2^	0.00
50 ppm fludioxonil	740	3.11 × 10^−2^	1.02 × 10^−7^
250 ppm fludioxonil	741	4.85 × 10^−2^	3.27 × 10^−7^

### Exposure of CAT to fludioxonil

At different concentrations of fludioxonil mixed with the CAT enzyme, its activities were measured and were given in Table 3. As fludioxonil concentrations increased, CAT was inhibited and its activity decreased. There were statistically significant differences between all determined values for the inhibited CAT, which interacted with the fludioxonil at the mentioned concentrations (*P* < 0.05, *n* = 3), and the control activity. The calculated per cent decrease of CAT enzyme activity after exposure to fludioxonil at concentrations of 10, 50, 100, 250 and 500 ppm, were as 20.0, 30.8, 42.8, 46.3 and 45.9, respectively.

**Table 3.** Effect of fludioxonil concentrations on catalase activity.

Fludioxonil concentration (ppm)	Activity ± standard error (U/mg)
0	711 ± 15 **a**
10	569 ± 12 **b**
50	492 ± 11 **c**
100	407 ± 9 **d**
250	382 ± 4 **d**
500	385 ± 7 **d**

Values are expressed as mean ± standard error (*n* = 3). In the table, “a, b, c and d” letters were used for differences of activity levels. There are statistical differences between the data which were shown with different letters (*p* ˂ 0.05).

### Inhibition type of fludioxonil to CAT

**Figure 3.**
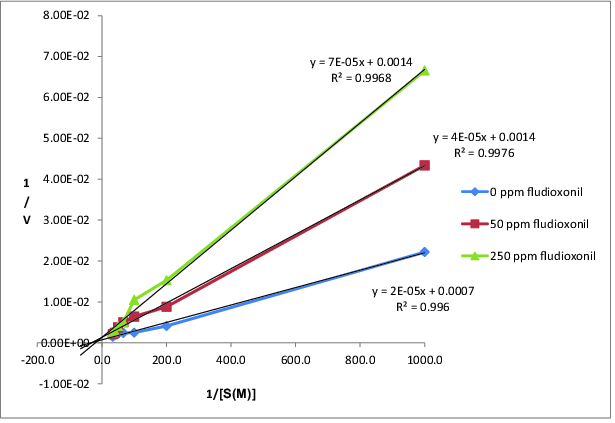
Lineweaver–Burk graph of CAT exposed with fludioxonil.

The activities of CAT were determined at different substrate concentrations of H_2_O_2_ (1, 5, 10, 15, 20, 25 and 30 mM) and at different concentrations of fludioxonil (50 and 250 ppm). Lineweaver–Burk graph of CAT was drawn by using the obtained results (Figure 3).

According to Figure 3, V_max_, K_m_ and K_i_ (inhibition constant) of CAT at different concentrations of fludioxonil were calculated and were shown in Table 2. The K_m_ values determined by us were near each other and we found decreased V_max_ values at the Lineweaver–Burk graph. Thus, it is suggested by us that fludioxonil acted on the activity of CAT via a non-competitive type of inhibition.

The optimum temperature for CAT determined by us was 35 °C. Çetinus et al. [15] reported same result for bovine liver CAT.[15] However, Tukel and Alptekin [14] indicated a maximum activity for CAT derived from the same source to be 25 °C.[14]

Karadag and Bilgin [16] treated cyprodinil and fludioxonil with a solution of copper-zinc superoxide dismutase (CuZnSOD), which was purified from human erythrocytes by diethylaminoethyl (DEAE)–cellulose chromatography and copper–iminodiacetic acid agarose chromatography.[16] They reported similar results for CuZnSOD. Cyprodinil inhibited CuZnSOD competitively and fludioxonil inhibited CuZnSOD non-competitively. In addition to this, other studies also examined the inhibition of CAT by pesticides. Li et al. [17] observed significant inhibition of CAT with prolonged exposure of propiconazole, a triazole fungicide, in the brain of the rainbow trout, *Oncorhynchus mykiss*.[17] When, Radice et al. [18] applied 0.3 and 0.4 mM, iprodione, a dicarboximide fungicide, to hepatocytes from rainbow trout (*Oncorhynchus mykiss*) they documented increasing both reactive oxygen species (ROS) and malondialdehyde (MDA) production and decreasing glutathione (GSH) content and CAT activity.[18] Toni et al. [19] applied different concentrations of the fungicide tebuconazole to common carp (*Cyprinus carpio*) and observed decrease in the activity of CAT at 1.19 mg/L.[19] Rehman et al. [20] studied the effect of deltamethrin in mice. They orally administered 2 doses of deltamethrin, 5.6 and 18 mg/kg body weight (bw), for 15 days. They observed suppressed CAT activity in liver and kidney of male Swiss albino mice.[20]

## Conclusions

According to our knowledge, this is the first study related to the effect of cyprodinil and fludioxonil on CAT enzyme activity. We observed inhibition of the CAT activity induced by these fungicides. We determined that cyprodinil inhibited CAT competitively and fludioxonil inhibited the enzyme non-competitively. Although cyprodinil molecules do not possess similarity to the molecules of hydrogen peroxide, we suggest that cyprodinil molecules generate radicals, which in turn compete with the hydrogen peroxide molecules to tie porphyrin heme (iron) groups.
